# Immunomodulatory Effects of Lidocaine: Mechanisms of Actions and Therapeutic Applications

**DOI:** 10.3390/ph19010134

**Published:** 2026-01-12

**Authors:** Jianwei Wu, Quanfu Chen, Zhiling He, Bin Yang, Zhenhua Dai, Feifei Qiu

**Affiliations:** 1 Section of Anesthesiology, The Second Affiliated Hospital of Guangzhou University of Chinese Medicine, Guangzhou 510006, China; 2 The Affiliated Traditional Chinese Medicine Hospital, Guangzhou University of Chinese Medicine, Guangzhou 510006, China; 3 Cardiovascular Department, The Affiliated Traditional Chinese Medicine Hospital, Guangzhou Medical University, Guangzhou 510000, China; 4 Department of Cardiovascular Sciences, College of Life Sciences, University of Leicester, Leicester LE1 9HN, UK; 5 Section of Immunology, The Second Affiliated Hospital of Guangzhou University of Chinese Medicine, 55 Nei Huan Xi Lu, College Town, Guangzhou 510006, China

**Keywords:** anesthetic, lidocaine, immunoregulation, immune cells, inflammatory disease, drug repurposing

## Abstract

Lidocaine, an amide-type regional anesthetic, has been an important medication in the field of anesthesia since its clinical approval. Recently, lidocaine has emerged as a powerful immunomodulatory agent beyond its classical anesthetic properties. This review has summarized the recent basic and clinical studies with sufficient evidence on the multifaceted effects of lidocaine on both innate and adaptive immune cells, including macrophages, neutrophils, eosinophils, basophils, natural killer (NK) cells, mast cells, dendritic cells (DCs), monocytes, and T lymphocytes. We have also detailed how lidocaine affects critical cellular processes, such as cellular polarization, cytokine production, phagocytosis, and apoptosis, through multiple signaling pathways, including NF-κB, TLR4/p38 MAPK, voltage-sensitive sodium channels, HIF1α, TGF-β/Smad3, AMPK-SOCS3, TBK1-IRF7, and G protein-coupled receptors. These immunoregulatory effects of lidocaine are dependent on its concentration, duration of action, and the microenvironment. The immunomodulatory actions of lidocaine may contribute to its potential therapeutic value in various settings of diseases, such as cancer, sepsis, acute lung injury, asthma, organ transplantation, ischemia–reperfusion injury (IRI), and diabetes. We propose that lidocaine can be repurposed as an immunomodulator for treating immune-mediated inflammatory diseases. However, future research should define optimal dosing strategies, validate its mechanisms of action in clinical trials, and explore its novel clinical applications as a complementary immunotherapy.

## 1. Introduction

Lidocaine, a regional anesthetic of the amide, is widely used in a clinical context. It was originally synthesized by Swedish chemist Nils Lofgren in 1942 and approved for clinical use in Sweden in 1948 [1,2]. Its chemical name is 2-(diethylamino)-N-(2,6-dimethylphenyl) acetamide with a molecular formula of C_14_H_22_N_2_O [3]. The primary mechanism of lidocaine involves blocking voltage-sensitive sodium channels of nerve cells, resulting in the suppression of the transient influx of sodium and nerve impulse transmission, exerting local anesthetic effects [4]. Due to its rapid action (1–3 min), moderate duration (1–3 h), potent efficacy, and strong tissue penetration [5], lidocaine has long been widely used in dentistry and minor or moderate surgeries. Since Gilbert et al. first proposed the use of intravenous lidocaine during surgery for postoperative analgesia in 1951 [6], a growing number of studies have shown that intravenous administration of lidocaine also induces analgesia [7,8], enhances neural protection [9], alleviates postoperative nausea and vomiting [10], and promotes the postoperative recovery of intestinal function. In recent years, mounting evidence has revealed that lidocaine possesses significant immunomodulatory properties. Its ability to influence a broad spectrum of immune cells offers promising clinical prospects: mitigating perioperative immunosuppression in cancer patients, tempering the cytokine storm in sepsis, or providing targeted anti-inflammatory therapy in chronic conditions such as asthma. Realizing this potential requires a systematic understanding of its cellular targets and molecular mechanisms.

**Scope of this Review**: This review comprehensively examines the immunoregulatory profile of lidocaine. We begin by detailing its effects on both innate immune cells (monocytes, macrophages, neutrophils, eosinophils, basophils, natural killer cells, mast cells, and dendritic cells) and adaptive immune cells (T lymphocytes), as supported by both in vitro and in vivo data. We then explore the therapeutic implications of these effects in the specific contexts of diseases, including sepsis, ALI, asthma, gastrointestinal inflammation, diabetes, and various cancers. Subsequently, we delve into the molecular signaling pathways mediating the effects of lidocaine. Finally, we discuss the limitations of current evidence, analyze the therapeutic window for clinical translation, propose specific application scenarios, and outline future perspectives.

## 2. Effects of Lidocaine on Innate Immune Cells

**Figure 1:**
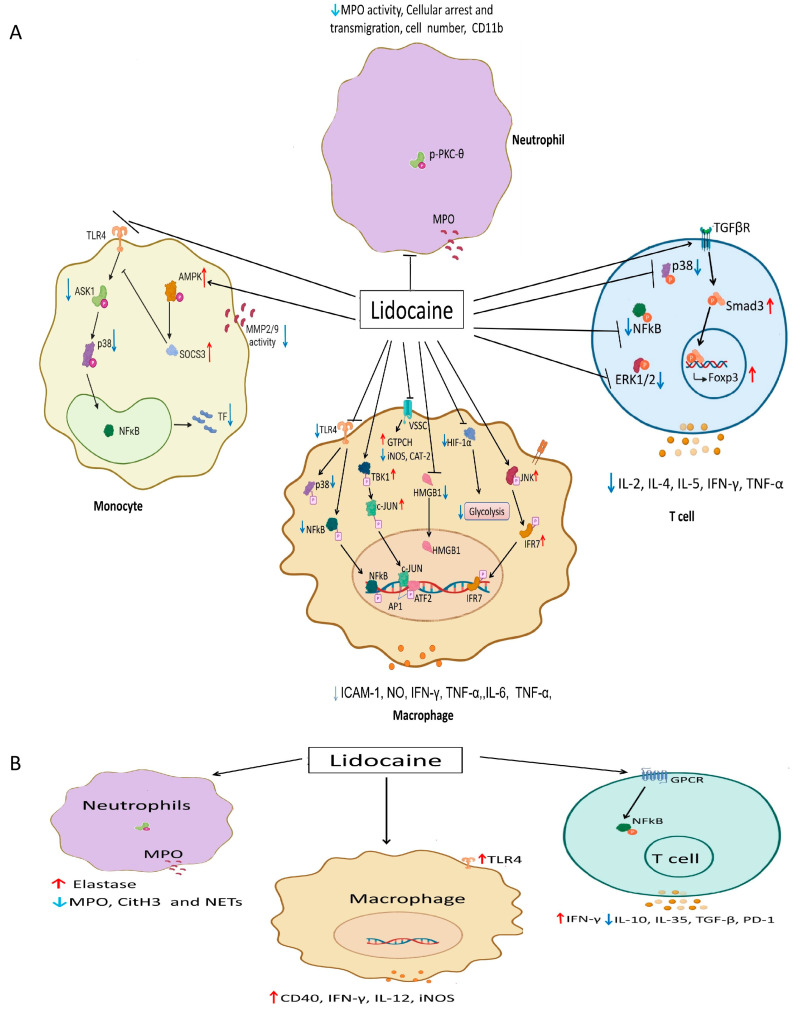
Signaling pathways regulated by lidocaine and the dual effects of lidocaine on immune cells in cancers and inflammatory disorders. Lidocaine exerts pro-inflammatory effects on immune cells in cancers, while it plays an immunosuppressive role in sepsis and inflammatory disorders. By interfering with NF-kB, TLR4/p38 MAPK, voltage-sensitive sodium channels, HIF-1α, TGF-β/Smad3, AMPK-SOCS3, TBK1-IRF7, and G protein-coupled receptor signaling pathways, lidocaine regulates pro-inflammatory or anti-inflammatory genes and matrix metallopeptidases in macrophages, monocytes, neutrophils, and T cells. (**A**) Immunomodulatory effects of lidocaine in inflammatory disorders. It focuses on anti-inflammatory/immunosuppressive effects). (**B**) Immunomodulatory effects of lidocaine in cancer. It focuses on pro-inflammatory/anti-tumor effects. The downward blue arrows indicate inhibition, while the upward red arrows denote stimulation/upregulation. (Abbreviations: ATF2, activating transcription factor 2; GPCR, G protein-coupled receptor; GTPCH, guanosine triphosphate cyclohydrolase I; TF, tissue factor; CAT-2, type-2 cationic amino acid transporter; HIF-1α, hypoxia-inducible factor 1 subunit alpha; HMGB1, high-mobility group box 1; IRF7, interferon regulatory factor 7; MMP2/9, matrix metallopeptidase 2/9; NFkB, nuclear factor kappa-B; SOCS3, suppressor of cytokine signaling 3; TGFβR, transforming growth factor beta receptor 1; TBK1, TANK binding kinase 1; Smad3, SMAD family member 3; VSSC, voltage-sensitive sodium channel).

Lidocaine can broadly regulate innate immune cells (Table 1, Table 2 and Table 3, Figure 1), as described below:

### 2.1. Monocytes

Mature monocytes from the bone marrow migrate into tissues [75] and further differentiate into macrophages or dendritic cells in response to local growth factors and cytokines [76], playing important roles in infection control, inflammation, and tissue repair. Although the inhibitory effects of lidocaine on monocytes have attracted attention, the mechanisms responsible for these effects remain unclear. It was reported that lidocaine not only profoundly dampened choline uptake and phosphatidylcholine biosynthesis [11], but also elicited chromatin condensation, DNA fragmentation, and cellular apoptosis, facilitating necrosis with the disturbance of membrane integrity in human monocyte-like U937 cells [12]. Under the stimulation of LPS, lidocaine dose-dependently restrained the expression of monocyte chemoattractant protein (MCP-1) and chemotaxis in THP-1 cells [13], and reduced the production of tissue factor (TF) and its activity in human blood monocytes [15]. In addition, the monocyte phagocytosis was inhibited by lidocaine [77]. Therefore, lidocaine exhibited suppressive effects on monocytes by inhibiting cell metabolism, chemokine secretion, procoagulant activity, and phagocytic function, thereby reducing inflammatory responses. However, further research is required to elucidate its specific mechanisms of action and potential clinical applications.

### 2.2. Macrophages

Macrophages differentiate from monocytes and participate in immune activation and tissue repair [78], exhibiting high plasticity and heterogeneity, while their polarization states and functional regulation play key roles in various diseases [79]. Lidocaine has shown significant potential in regulating the function of macrophages [28,32,36] and their inflammatory response [23,80] through altering cellular polarization, phagocytosis, and the production of cytokines or superoxide anion.

#### 2.2.1. Macrophage Polarization

The effects of lidocaine on macrophage polarization are not yet fully understood. However, some studies showed that it promoted the formation of pro-inflammatory, anti-tumor M1 macrophages. In coculture with breast cancer cells, lidocaine (1 mM) can repolarize macrophages from a pro-tumorigenic M2 phenotype (CD204+CD206+) to an anti-tumor M1 phenotype (F4/80+CD86+), leading to the downregulation of Arg-1 and the upregulation of iNOS [16]. In addition, in tumor-infiltrating immune cells (TIICs) from gastric cancer, lidocaine treatment (0.25–1.5 mM) enhanced M1-related CD40, IFN-γ, and IL-12 while reducing IL-10, TGF-β, and IL-35 production, but these effects could be reversed by TLR4 inhibitor, indicating that lidocaine facilitates the polarization towards the M1 macrophage phenotype via the promotion of the toll-like receptor 4 (TLR4) signaling [24]. Nevertheless, in healthy donor cells, it promoted the expression of anti-inflammatory cytokines, suggesting that its effects may vary depending on the microenvironment [24].

#### 2.2.2. Cytokine Production

Lidocaine can obviously influence the production of various inflammatory cytokines by macrophages. For example, studies revealed that lidocaine treatment (300 µM) augmented the expression of IFNα4 in virus-infected macrophages through activating TBK1-IRF7 and JNK-AP1 signaling pathways [21]. Lidocaine could also downregulate the expression of anti-inflammatory cytokines (Arg-1, TGF-β, IL-10, TGF-β, and IL-35) while upregulating that of pro-inflammatory cytokines (NO, IFN-γ and IL-12) in macrophages in the tumor microenvironment [16] or in a Leishmania-infected state [25]. On the other hand, other studies indicated the opposite impacts of lidocaine on the expression of inflammatory cytokines in macrophages. Lidocaine dose-dependently reduced the levels of NO metabolites (nitrite and nitrate) by decreasing inducible NO synthase (iNOS) protein production in macrophages challenged by LPS [17,22]. Furthermore, exposure to different doses of lidocaine also led to a decrease in production of inflammatory cytokines (IL-1β, IL-6, IL-8, TNF-α, and HMGB1) in both human [25,26,31] and murine macrophages [18,27,29,62] under stimulation. Beyond modulating classical polarization markers, lidocaine may regulate macrophage function by influencing tissue repair and regeneration. Recent research has highlighted that the P2X7R/NLRP3 inflammasome axis in macrophages critically suppresses enthesis regeneration by modulating inflammatory and metabolic cross-talks in stem cells [81]. This underscores the potential significance of the documented inhibitory effects of lidocaine on the NLRP3 inflammasome [27] in not only controlling inflammation but also potentially facilitating a pro-regenerative microenvironment.

#### 2.2.3. Phagocytic Function

The phagocytic function of macrophages is crucial for clearing pathogens. Macrophages recognize and bind specific antigens on the surface of foreign substances through toll-like receptors (TLRs), engulf them to form phagosomes within the cell, and then degrade or digest the foreign particles using intracellular lysosomal enzymes [82]. A previous study showed that lidocaine (10 mM) treatment induced antibody-dependent phagocytosis of sheep red blood cells by macrophages through activation of Fc receptor functions [30]. Moreover, it strengthened the phagocytic capacity of Kupffer cells in diabetic mice [62]. Nevertheless, early experiments performed by others revealed the suppressive roles of lidocaine in the phagocytic function of macrophages. It was reported that lidocaine treatment led to less uptake of Herpetomonas samuelpessoai, followed by more cytoplasmic vacuoles in macrophages [83]. It suppressed the macrophage phagocytosis of opsonized zymosan [34] and latex beads [19]. In addition, lidocaine (5 mM) suppressed the digestion of immune complexes and the degradation of immunoglobulin aggregates by macrophages [33].

#### 2.2.4. Production of Superoxide Anion

Lidocaine decelerated intracellular pH recovery in a dose-dependent manner. Lidocaine (2.5 mM) suppressed the exchange of Na+/H+, decreased the V-ATPase-regulated component of intracellular pH recovery, and finally suppressed the superoxide production [35].

#### 2.2.5. Metabolic Reprogramming

Lidocaine suppresses LPS-induced glycolysis in macrophages by downregulating HIF1α and its target genes GLUT1 and HK2. This metabolic shift away from glycolysis has contributed to reduced pro-inflammatory cytokine production and may underlie its anti-inflammatory effects on sepsis [29].

The modulation of macrophage function by lidocaine involves a mechanism of integrated interplay: (1) Channel blockade. The inhibition of voltage-sensitive sodium channels (VSSC) is a pivotal upstream event. The effects of lidocaine on iNOS and pro-inflammatory pathways can be reversed by VSSC activators, suggesting that the altered membrane potential or sodium influx initiates downstream signaling cascades [17,20]. (2) Receptor and kinase inhibition. This initial signal leads to the suppression of key surface receptors (e.g., TLR4) and intracellular kinases (e.g., IKK, p38 MAPK), resulting in decreased activation of transcription factors such as NF-κB and AP-1. (3) Metabolic reprogramming. Lidocaine suppresses HIF-1α-driven glycolysis in activated macrophages [29]. By limiting this major energy source, it curtails the metabolic capacity required for sustained pro-inflammatory responses, thus adding a crucial metabolic layer to its anti-inflammatory action.

Taken together, lidocaine regulates macrophage function via the regulation of polarization status, inflammatory cytokine secretion, phagocytosis, and superoxide anion. These effects make it a potential therapeutic drug for treating inflammatory diseases, infections, and cancers. Nevertheless, further study is necessary to identify the specific mechanisms underlying the effects of lidocaine, especially its mechanistic impacts on macrophage function at different concentrations and timings.

### 2.3. Neutrophils

As a crucial part of blood cells, neutrophils serve as the first line of our defense against pathogens, constantly protecting us from diseases [84]. Moreover, they participate in both immune regulation and inflammatory responses. Increasing studies indicated that lidocaine had an impact on neutrophils [37]. Intravenous bolus injection of lidocaine decreased the count of neutrophils in the bronchoalveolar lavage fluid (BALF) of canine lung allograft [63], the horse colonic mucosa after ischemia for 1 h [64], and the blood from mice with acute lung injury [14]. In addition, inhaled lidocaine suppressed the accretion of neutrophils in BALF and peribronchial tissue triggered by ovalbumin, thus alleviating airway hyperreactivity in a murine model of asthma [58].

#### 2.3.1. Chemotaxis and Migration

The integrin CD11b/CD18, composed of α (CD11b) and β (CD18) subunits, binds to ICAM-1 on the endothelial cell surface, and then facilitates neutrophil migration to inflammatory sites [85]. The activation of CD11b/CD18 promoted the expression of inflammatory factors, including IL-8 and TNF-α, exacerbating inflammatory responses [86]. Intravenous bolus injection of lidocaine dampened the CD11b expression of neutrophils in peripheral blood from recipient dogs with lung allograft [63]. It also reduced the production of CD11b, CD18, and ICAM-1 in neutrophils exposed to plasma from patients with tourniquet-induced ischemia and reperfusion [38]. Similarly, in the presence of the platelet-activating factor, porcine neutrophils treated with lidocaine (1–100 μM) exhibited significantly less CD11 b/c expression than control neutrophils did [44].

It was demonstrated that lidocaine inhibited both chemotaxis and phagocytosis of in vitro neutrophils isolated from healthy volunteers [39]. A clinical trial conducted by Berger et al. also showed that lidocaine treatment in septic patients suppressed neutrophil arrest induced by chemokines and dampened cellular transmigration through repressing the protein kinase C-θ without changing the selectin-regulated slow rolling, suggesting an inhibitory effect of lidocaine on neutrophils during sepsis [68]. However, Cook et al. [45] observed that lidocaine at higher concentrations promoted equine neutrophil migration or adhesion induced by IL-8 alone or the combination of IL-8 and LTB in vitro.

#### 2.3.2. Neutrophil Extracellular Traps (NETs)

Upon encounter with pathogens, neutrophils undergo a programmed cell death with web-like structures, which are composed of decondensed chromatin and intracellular granular proteins, known as NETs, thereby confining pathogens to localized infection areas and eliminating captured microorganisms [87]. NETs can either regulate immune responses or enhance immune reactions [88]. High levels of NETs are related with increased recurrence and metastasis in cancers [89]. Several studies showed that lidocaine significantly reduced NET formation. The intravenous administration of lidocaine during operation led to a decrease in plasma levels of NETs biomarkers (citrullinated histone H3 and myeloperoxidase) in patients with breast, lung, prostate, and pancreatic cancers [69,70,71,72]. We propose that lidocaine may inhibit NETosis by modulating intracellular calcium flux, a critical trigger for the enzyme PAD4 [90], or by suppressing mitochondrial ROS production [88,89].

#### 2.3.3. Respiratory Burst of Neutrophils

The respiratory burst of neutrophils is a process in which neutrophils produce a large amount of reactive oxygen species in response to pathogens, thus clearing invading microorganisms [91]. Lidocaine lessened the oxidative burst, reduced ATP concentration in neutrophils, and lowered mitochondrial transmembrane potential while promoting mitochondrial structural changes and cellular apoptosis [41,42]. Moreover, lidocaine addition significantly decreased the secretion of reactive oxygen species containing O_2_^−^, H_2_O_2_, and OH^−^ in isolated human [39,43] and porcine neutrophils [44].

Taken together, lidocaine regulates neutrophil functions through multiple pathways, including the inhibition of migration, chemotaxis, NET formation and respiratory burst, therefore exerting anti-inflammatory effects. These effects have potential clinical values in alleviating ischemia–reperfusion injury, sepsis, and postoperative inflammation. However, attention should be paid to concentration-dependent differences and the complex impact of lidocaine on immune function.

### 2.4. Eosinophils

Eosinophils play important roles in immunologic and allergic reactions [92]. Eosinophils have strong killing power against parasitic infections. They can destroy parasites through phagocytosis, releasing granule enzymes, and producing oxygen-free radicals [93]. Recent studies have demonstrated that lidocaine has significant immunomodulatory roles in regulating eosinophil activity and survival. These effects are mainly manifested in the following aspects.

The number of blood eosinophils was significantly decreased in patients with nebulized lidocaine compared to that in patients with placebo [73]. Similarly, inhaled lidocaine reduced the number of eosinophils in BALF and the peribronchial tissue of mice with OVA-induced airway hyperreactivity [58]. In the presence of different stimulus, such as IL-5, IL-3, or GM-CSF, lidocaine inhibited the survival of eosinophils and reduced the eosinophil count, accompanied by a decreased production of neurotoxin, superoxide, and peroxidase in vitro [46,47,48]. In addition, lidocaine alleviated airway response to inhaled methacholine in asthmatic cats without an impact on eosinophilia in BALF [94]. Lidocaine exerts immunomodulatory effects by reducing eosinophil counts and inhibiting their survival and activation, indicating its potential therapeutic value for allergic diseases (such as asthma). However, more studies are needed to identify its mechanisms of action and clinical effects.

### 2.5. Basophils

Basophils, less than 1% of circulating white blood cells, have similar functions to mast cells, such as expressing high-affinity receptors for IgE and releasing histamine upon activation [95]. Basophils are associated with parasite infection, inflammation, and allergic immune responses. When IgE antibodies of basophils bind to antigens, the cells undergo degranulation and secrete inflammatory mediators, such as histamine [96]. A previous investigation showed that lidocaine (1–10 mM) dose-dependently reduced the histamine secretion and intracellular calcium concentration of murine basophils in response to stimulation in vitro [49]. However, the effects of lidocaine on basophil activation were recently examined via baseline activation testing (BAT) in healthy volunteers. Garcia-Nunez et al. demonstrated that lidocaine did not significantly induce basophil activation in five healthy subjects [97]. This finding indicates that lidocaine may have an impact on basophil activity only under certain conditions.

### 2.6. Natural Killer (NK) Cells

NK cells, originating from bone marrow lymphoid stem cells, can directly recognize and attack tumor, virus-infected, and other abnormal cells, secreting cytotoxin containing perforin and granzymes and then leading to cell death [98,99]. This unique killing mechanism makes NK cells the first line of our body’s defense. In a clinical study, intravenous injection of lidocaine during operation significantly increased the frequency of NK cells in the blood of patients receiving primary breast tumor resection [74]. However, another in vitro study revealed that lidocaine did not influence the percentage of NK cells and their cytotoxic subpopulation (CD56^dim^CD16^bright^) in peripheral blood mononuclear cells (PBMCs) from healthy volunteers [100]. Therefore, the impacts of lidocaine on NK cells remain controversial, as its effect may depend on the concentration, experimental conditions, and the individual research subjects.

Multiple studies have shown that lidocaine can enhance the cytotoxicity of NK cells at clinically relevant concentrations. Ramirez et al. found that lidocaine (0.01–50 μM) augmented the cytotoxicity of NK cells against cancer cells in vitro, such as K562, THP-1, and OCI-AML3 cells, through increasing granzyme B and perforin [50]. In addition, lidocaine augmented the expression of activating receptors NKG2D on NK cells from healthy donors and their cytotoxicity against cancer cells, including SKOV3, KRIB, and PATC53 cells. Similar effects of lidocaine were also observed on NK cells derived from patients receiving oncological surgery [51].

On the other hand, lidocaine may inhibit the activity of NK cells under high concentrations or specific conditions. The intratracheal instillation of lidocaine (1%) suppressed the activity of NK cells in normal rat lung lavage [65]. Lidocaine also downregulated the in vitro activity of NK cells derived from normal human peripheral blood [52].

Taken together, the effects of lidocaine on NK cells are redundant and may be dose- and context-dependent, leading to apparent contradictions in the literature: (1) Activating effects. At low or clinical concentrations (0.01–50 μM), lidocaine may enhance NK cell cytotoxic function against tumor cells (e.g., K562, SKOV3 cell lines) by upregulating activation receptor NKG2D and increasing granzyme B and perforin release [50,51]. Intravenous administration of lidocaine during surgery increases peripheral NK cell frequency in breast cancer patients [74], showing its potential therapeutic value in anti-tumor immunity. (2) Inhibitory roles. High concentrations/local application (e.g., intratracheal instillation) may suppress NK cell activity [52,65]. The inconsistent impacts of lidocaine on NK cells could result from different concentrations, the route of administration, and various experimental models. The therapeutic window for immunomodulation appears to be within clinically achievable systemic concentrations. Thus, further research is warranted to define the mechanisms underlying this discrepancy.

### 2.7. Mast Cells

Mast cells are divided into connective tissue mast cells (CTMCs) and mucosal mast cells (MMCs) [101,102], playing a role in immune responses via directly phagocytosing pathogens and secreting cytokines or chemokines [103]. CTMCs release tryptase and chymase and are involved in antigen presentation, while MMCs secrete histamine and participate in mucosal immunity [102]. Animal studies revealed that lidocaine significantly attenuated mast cell hyperplasia in mucosa, improved rat colitis induced by trinitrobenzenesulfonic acid (TNBS) [66], and diminished mast cells at the wound site of rats [67]. Moreover, low concentrations (lower than 30 mM) of lidocaine in vitro dose-dependently restrained the Ca^2+^ influx and histamine release from murine mast cells upon different stimulation, including ionophore A23/187, compound 48/80, and ConA [49,54,55,104], suggesting that blocking calcium channels by lidocaine contributed to the suppression of calcium-dependent histamine release [105]. Meanwhile, high concentrations (60 to 100 mM) of this drug facilitated histamine secretion and cell lysis, indicating that this effect was dependent on drug concentration and pH value [53]. Collectively, lidocaine has a bidirectional regulatory effect on mast cells. It inhibits histamine release by blocking calcium channels at low concentrations, hence exerting anti-inflammatory and anti-allergic effects. However, it may disrupt cell membrane stability, leading to increased histamine release at high concentrations. Altogether, lidocaine with reasonable doses may be used to treat mast cell-mediated inflammatory diseases, such as colitis and allergic reactions.

### 2.8. Dendritic Cells (DCs)

Dendritic cells have a significant impact on the initiation, regulation, and maintenance of immune responses through recognizing molecular patterns related to pathogens and via modulating T cell responses [106]. Lidocaine reduced the production of pro-inflammatory cytokines (IL-6, TNFα and IL-12) by bone marrow-derived DCs under the stimulation of TLR ligands containing LPS, poly (I:C), and R837, suggesting their impaired maturation [56]. In addition, lidocaine selectively curbed DC-mediated Th1 response in vitro and in vivo, without affecting Th2 and Th17 cells [56]. Lidocaine also indirectly inhibited neuroimmune cross-talk. Due to its suppression of calcitonin gene-related peptide (CGRP) release from nerves, lidocaine (0.05, 0.1 mM) reduced IL-23 release by CGRP receptor-expressing dendritic cells cocultured with dorsal root ganglion neurons, suggesting that this drug dampens IL-23 production and thus ameliorates psoriasis via preventing neurogenic CGRP signaling from being transmitted to DCs [107].

These results indicate that the mechanisms underlying the effects of lidocaine on DCs are complicated, and more studies are needed to elucidate its full effects. Thus, lidocaine can directly reduce the pro-inflammatory cytokine secretion by TLR-activated DCs and may selectively inhibit DC-mediated Th1 responses. On the other hand, it can indirectly reduce IL-23 release in DCs by blocking neurogenic CGRP signals, indicating its potential application as an immunomodulator for the treatment of inflammatory and autoimmune diseases.

## 3. Effects of Lidocaine on Adaptive Immune Cells (T Lymphocytes)

T lymphocytes, identified by the surface marker CD3, mediate adaptive immunity [108]. T cells are divided into several subpopulations according to their phenotypes and functions, including CD4+ helper T (Th) cells, regulatory T (Treg, often CD4+CD25+FoxP3+) cells, and cytotoxic T cells (CD8+, CTLs) [108]. Th cells eliminate pathogens via secreting various cytokines that act on other immune cells [109]. Treg cells prevent T cells from excessive immune responses and unnecessary damage to self-tissues and organs [110]. CTLs clean up infected target cells through producing perforin, granzymes, and other cytotoxic granules [111]. Lidocaine treatment apparently led to a rise in the percentage of CD3+ and CD4+ T cells in patients receiving primary breast tumor resection, whereas the percentage of CD8+ T cells was not significantly changed (*p* > 0.05) [74]. Thus, lidocaine regulates not only the number of T cells, but also their cytokine production, cellular proliferation/differentiation, and apoptosis (Table 1 and Table 2 and Figure 1).

### 3.1. Cytokine Production and Functional States

Available data have shown that lidocaine attenuates pro-inflammatory cytokine production (IL-2, IL-5, INF-γ and TNF-α) by CD3+ T cells from human blood and mouse Peyer’s patch [57,59]. Further, it was demonstrated that lidocaine decreased the expression of IL17E mRNA in both lesional skin and PBMCs from atopic dermatitis (AD) patients, while enhancing mRNA expression of cytokines (IFN-γ, IL4, and IL17A) in PBMCs. Similar results were observed in murine AD models. Lidocaine elevated the IFN-γ mRNA level while decreasing mRNA levels of IL-4 and IL-17E in lesional skin of AD mice [60]. Lidocaine also dampened IFN-γ expression in CD8+ PBMCs from healthy donors while enhancing cytokines (IL-10, TGF-β, IL-35) in CD4+CD25+ Treg cells [24]. On the other hand, in the tumor microenvironment (e.g., tumor-infiltrating immune cells in gastric cancer), lidocaine demonstrated a unique ability to increase IFN-γ secretion in CD8+ T cells through G protein-coupled receptor signaling and NF-κB activation, while reducing immunosuppressive cytokines (IL-10, TGF-β, IL-35) by CD4+CD25+ Treg cells, thereby facilitating an anti-tumor response [24]. These findings suggest it can potentially reverse aspects of T cell exhaustion in tumors while suppressing general inflammation.

### 3.2. Proliferation and Differentiation

Several experiments indicated that lidocaine (1–2 mM) attenuated the proliferation of mouse and human T cells in vitro [57,58,59]. Nevertheless, Berkeley et al. demonstrated that T cell proliferation in vitro was not affected by lidocaine (0.5 mM) [112]. Regarding cellular differentiation, lidocaine not only indirectly restrained the differentiation of Th1 cells in vitro through suppressing DC activation induced by OVA [56], but also promoted Treg cell differentiation in vitro via the Smad3/TGF-b signaling pathway [60].

### 3.3. Apoptosis

Lidocaine promoted the apoptosis of Jurkat and mouse T cells in vitro [58,61]. In addition, Matsuo et al. reported that lidocaine (1 mM) facilitated the apoptosis of CD4+ T cells via activating caspase-3 and caspase-9 while reducing BCL-2. Their in vitro experiments showed that the frequency of apoptotic cells was remarkably higher in CD4+ T cells treated with lidocaine than that in control CD4+ T cells, while the expressions of IL-5 and IFN-γ were dampened by lidocaine [113].

In summary, these findings suggest that lidocaine exerts immunomodulatory effects by regulating T cell subsets, cytokines, proliferation and differentiation, and apoptosis. However, its efficacy may vary depending on the concentration, cell type, and pathological condition (Table 1, Table 2 and Table 3).

## 4. Immunomodulatory Effects of Lidocaine on Sepsis and Other Allergic/Inflammatory Diseases

Accumulating evidence suggests that the therapeutic potential of lidocaine extends far beyond pain management, with demonstrated efficacy in modulating immune responses across various disease states. By integrating data from both preclinical models and clinical trials, we provided a detailed understanding of how lidocaine influences immune cell function to exert therapeutic effects mainly on inflammatory diseases (Figure 1).

### 4.1. Sepsis

Lidocaine exhibited significant immunomodulatory effects in the context of sepsis by targeting neutrophil function. It specifically inhibited PKC-θ, thus blocking neutrophil arrest and transendothelial migration while preserving selectin-mediated rolling [68]. This selective action maintained immune surveillance while mitigating harmful inflammatory responses. In addition, lidocaine suppressed HIF1α-mediated glycolysis in macrophages, reducing production of pro-inflammatory cytokines and LPS-induced inflammation [29]. These actions support the future investigation of lidocaine as an adjunctive infusion in septic patients to alleviate organ damage without compromising host defense.

### 4.2. Acute Lung Injury

In sepsis-induced acute lung injury (ALI), lidocaine activated the AMPK-SOCS3 axis to suppress the TLR4/ASK1/TF pathway [14], thus reducing TF accumulation, fibrin deposition, and pulmonary thrombosis while decreasing MMP-2/9 activity [14]. The resultant effects included a reduction in lung edema and neutrophil infiltration and an improvement of survival rates in ALI models. The anti-inflammatory effects on the pulmonary endothelium, an important site of injury in ALI, are further supported by broader mechanisms involving the NF-κB pathway. For instance, the activation of Sirtuin 6 (SIRT6) was shown to relieve LPS-induced inflammation in human lung microvascular endothelial cells through inhibiting NF-κB activation [114], highlighting a key molecular target within the vascular compartment.

### 4.3. Reperfusion Injury

The effects of lidocaine on neutrophil function can be extended to ischemia–reperfusion injury, as demonstrated in canine lung allograft models [63]. This drug maintained basal CD11b/CD18 expression and reduced MPO activity, thereby improving gas exchange and reducing neutrophil infiltration into BALF [63]. These findings indicate potential applications of lidocaine in organ transplantation and other conditions characterized by reperfusion injury.

### 4.4. Asthma

In murine asthma models, inhaled lidocaine dramatically reduced hallmark features of allergic airway diseases through multiple mechanisms. This drug suppressed GATA-3 expression and reduced the secretion of IL-4, IL-5, and IL-13 [58], resulting in reduced eosinophil-mediated inflammation, airway hyperreactivity, mucus hypersecretion, and airway remodeling. Clinical trials also showed that lidocaine significantly improved FEV1, reduced nighttime awakenings and symptoms, decreased blood eosinophil counts, and reduced glucocorticoid dependence in asthmatic patients receiving nebulized lidocaine [73]. Nebulized lidocaine could be considered for severe and steroid-dependent asthma.

### 4.5. Gastrointestinal Inflammation

Lidocaine showed beneficial effects in experimental colitis through blocking neuropeptide, inhibiting substance P-mediated granulocyte infiltration, and reducing mucosal mast cell hyperplasia [66]. These effects were evident with both intrarectal and subcutaneous administration of lidocaine. Equine models of intestinal manipulation also revealed tissue-specific effects of lidocaine on gastrointestinal inflammation. Lidocaine significantly reduced COX-2 production in circular muscle layers of manipulated jejunum without consistently suppressing neutrophil infiltration across all tissue types [64]. Additionally, it preserved transepithelial resistance while reducing mannitol flux in manipulated intestine, suggesting protective effects on intestinal barrier function during surgical manipulation [64].

### 4.6. Diabetes

Lidocaine corrected the dysfunction of Kupffer cells (KCs) in diabetic models by restoring phagocytic capacity and reducing ICAM-1-mediated granulocyte recruitment [62]. These effects occurred specifically in diabetic mice, with no significant changes observed in non-diabetic controls, suggesting a targeted correction of diabetes-associated immune dysfunction [62]. This immunomodulation of hepatic KCs may be particularly relevant given that patients with noncancer end-stage liver diseases exhibit a distinct and dysregulated immune and inflammatory microenvironment [81]. It may represent a compelling clinical research direction to investigate whether lidocaine can ameliorate similar immune dysfunction in the context of diabetes-associated liver diseases.

Taken together, the existing evidence has demonstrated that the immunomodulatory effects of lidocaine are both context-dependent and tissue-specific. In cancer models, it most prominently affects TAM reprogramming and NET inhibition as well as T cell function, while in sepsis and inflammatory disorders, its immunosuppressive effects predominate (Figure 1). These results indicate that the effects of lidocaine are complex, with a notable tissue and disease specificity.

## 5. Effects of Lidocaine on Cancers

Lidocaine exhibits significant antimetastatic effects in breast cancer models through multiple immunomodulatory mechanisms. When combined with general anesthetics, such as propofol or sevoflurane, lidocaine reprogrammed tumor-associated macrophages (TAMs) from the M2 phenotype to the tumoricidal M1 phenotype, resulting in decreased epithelial–mesenchymal transition (EMT) and reduced metastatic potential [16]. Additionally, lidocaine significantly attenuated the formation of NETs in lung and prostate cancer patients, as evidenced by reduced MPO and CitH3 levels, thereby disrupting the pro-metastatic niche [70,71]. In breast cancer patients undergoing tumor resection, the intravenous infusion of lidocaine maintained NK cell activity and CD4+/CD8+ T cell ratios, potentially improving postoperative immunosuppressive status [74].

In gastric cancer, lidocaine exhibited distinct immunomodulatory properties by enhancing CD8+ T cell cytotoxicity through the GPCR/NF-κB-mediated upregulation of IFN-γ [24]. Simultaneously, it promoted an M1-like phenotype in tumor-infiltrating macrophages via TLR4 activation, creating an unfavorable microenvironment for tumor growth. This drug differently modulated cytokine production in peripheral PBMCs versus TIICs. While it increased anti-inflammatory cytokines in CD4+CD25+ and CD14+ PBMCs from healthy donors, it enhanced IFN-γ and IL-12 expression in CD8+ and CD14+ TIICs from gastric cancer patients [24]. This selective immunomodulation enhanced anti-tumor immune responses without compromising general immunity.

## 6. Molecular Signaling Pathways: An Integrated Network

The immunomodulatory effects of lidocaine on various immune cells converge on several key signaling hubs, forming an interconnected network rather than isolated pathways. The context (cell types, disease state, and concentrations) determines which nodal points in this network are most significantly engaged, explaining its pleiotropic and dual effects. These findings suggest that lidocaine may have broad therapeutic potentials in regulating immune responses in diverse inflammatory and autoimmune diseases. However, further studies are needed to fully understand the mechanisms underlying the actions of lidocaine on immune cell function and to better evaluate its safety and efficacy in a clinical context.

### 6.1. NF-κB and TLR4/MAPK Signalings

TLR4 recognizes different pathogen-associated molecular patterns (PAMPs) and then initiates downstream signaling through a MyD88-dependent pathway, including NF-κB and MAPK signaling pathways [115,116,117,118,119,120], which exert regulatory effects on cell survival and death, immune responses, and inflammatory reactions, thus relating to inflammatory or autoimmune diseases [119]. Lidocaine obviously diminishes the expression of TLR-4 and the downstream activation of NF-kB [59], ERK, and p38 MAPK in T cells and macrophages upon stimulation [18,20], resulting in a decline in the expression of pro-inflammatory genes (e.g., IL-2 and TNF-α) [24,59]. This axis is crucial in sepsis, ALI, and general inflammation. On the other hand, treatment with NF-κB inhibitors can reverse the effects of lidocaine on the expression of IFN-γ and PD-1 in CD8+ TIICs, while TLR4 inhibitors counteract the effects of lidocaine on the expression of IL-2 in CD14+ TIICs [24]. Therefore, lidocaine plays dual roles in regulating TLR4 and p38 MAPK signal pathways, depending on disease settings.

### 6.2. Voltage-Sensitive Sodium Channels (VSSCs)

VSSCs are a critical ion channel protein involved in the generation of membrane potentials and bioelectrical signaling [121] and a canonical target of lidocaine [122]. It was demonstrated that the effects of lidocaine on the expression of iNOS, CAT-2, and GTPCH in LPS-activated macrophages were dampened by veratridine, an activator of sodium ion channels [17]. The inhibitory effects of lidocaine on the expression of TLR-4, NF-kB, ERK, and p38 MAPK were also mitigated by veratridine [20]. The blockade of VSSCs is implicated as an upstream event that initiates many immunomodulatory cascades, including the inhibition of NF-κB/MAPK in macrophages and the regulation of calcium flux in mast cells or neurons [20,49]. Thus, lidocaine can regulate immune responsiveness through VSSCs.

### 6.3. HIF1α Signaling

The protein hypoxia-inducible factor-1 (HIF-1) is a crucial transcriptional factor activated under hypoxic or inflammatory conditions and a key molecule regulating glycolysis [123]. Lidocaine reduced the expression of HIF1α in LPS-stimulated macrophages and suppressed glycolysis and glycolytic capacity [29], limiting the metabolic fuel for activated immune cells. Hence, lidocaine exerts a suppressive effect on HIF1α signaling.

### 6.4. TGF-β/Smads Signaling

TGF-β/Smad signal pathways in immune cells are involved in immune regulation, cell differentiation, and apoptosis control [124,125,126]. Lidocaine was reported to significantly activate the TGF-β/Smad3 signaling pathway in T cells in vitro [60], leading to an increase in FoxP3 expression and the frequency of Treg cells.

### 6.5. AMPK-SOCS3 Signaling Pathway

The AMPK-SOCS3 signaling pathway is involved in energy metabolism, immune regulation, and cytokine signaling control [127,128,129]. THP1 cells treated with lidocaine elevated their expression of p-AMPK and SOCS3; these effects could be prohibited by AMPK inhibitor, demonstrating that lidocaine activates the energy-sensing AMPK-SOCS3 axis [14].

### 6.6. TBK1-IRF7 and JNK-AP1 Signaling

Both TBK1-IRF7 and JNK-AP1 signalings are important for IFNα production. Lidocaine enhanced the phosphorylation of TBK1 and IRF7 as well as the activation of JNK and c-Jun in uninfected or infected macrophages, contributing to increased IFNα4 production and antiviral potential [21]. These results indicated that lidocaine augmented the antiviral capacity of macrophages through TBK1-IRF7 and JNK-AP1 pathways.

### 6.7. G Protein-Coupled Receptor Signaling

G protein-coupled receptor (GPCR) signaling is an important mechanism by which cells respond to external stimulus. Wu et al. revealed that IFN-γ expression induced by lidocaine in CD8+ TIICs was counteracted by GPCR inhibitors [24], suggesting that lidocaine regulates IFN-γ expression in CD8+ TIICs via GPCR signaling.

## 7. Clinical Translation: Dosing, Scenarios, and Challenges

**Therapeutic window analysis:** A critical consideration is to align immunomodulatory doses with clinical safety. Typical analgesic plasma concentrations of systemically administered lidocaine range from 1 to 5 µg/mL (∼4 to 20 μM). Notably, many in vitro immunomodulatory effects (e.g., enhanced NK cytotoxicity at 0.01–50 μM, monocyte suppression at 0.3–300 μM, cytokine modulation, etc.) occur within or near the ranges, suggesting its clinical feasibility [12,50,74]. Higher concentrations used in some in vitro studies (>0.1 mM) may be relevant for local/topical applications (e.g., inhalational and intra-articular) but are not practical for systemic use. This analysis underscores that many immunomodulatory effects of lidocaine are attainable at safe, established doses.

**Proposed clinical integration scenarios:** (1) Oncological surgery: Perioperative intravenous infusion (e.g., 1.5 mg/kg bolus followed by 1–2 mg/kg/h) to mitigate surgery-induced immunosuppression, potentially improving long-term outcomes by preserving NK/T cell function and reducing NETs [70,74]. (2) Critical care: As an adjuvant in sepsis to dampen excessive neutrophil activation and cytokine storm, possibly improving organ function [14,68]. (3) Chronic inflammatory disorders: Nebulized lidocaine for severe, steroid-dependent asthma [73]; topical/rectal formulations for localized conditions like ulcerative proctitis or plaque psoriasis [66,107]. (4) Regional anesthesia: Choosing lidocaine-based peripheral nerve blocks or epidurals may offer immunological advantages over other agents in patients undergoing major surgery.

**Current challenges:** It remains challenging to standardize dosing, identify predictive biomarkers of response, and conduct large-scale outcome trials, but they are certainly worth pursuing for the next steps.

## 8. Limitations and Future Perspectives

While the preclinical evidence is compelling, significant gaps remain. There are some limitations in the recent studies on the immunoregulatory effects of lidocaine: (1) Preclinical focus: The vast majority of the studies concerning the immunoregulatory and anti-inflammatory effects of lidocaine in the context of inflammation have been conducted in small animal models, whereas there are only a few clinical studies in this aspect. (2) Heterogeneity: Clinical trials are often small, with variable dosing regimens and routes (*iv*, inhalational, and local), and short follow-up. (3) Long-term data: Long-term immunomodulatory safety and impacts on infection risk are unknown.

Future directions of studies should be focused on the following: (1) conducting large-scale, randomized controlled trials (RCTs) to validate efficacy in specific conditions (e.g., perioperative cancer immunotherapy and severe asthma, etc.); (2) defining optimal dosing and routes for immunomodulation versus anesthesia; (3) exploring combinatorial therapies (e.g., lidocaine with immune checkpoint inhibitors for cancer therapy); (4) investigating long-term immunoregulatory outcomes; and (5) utilizing pharmacogenomics to identify patient subgroups that most likely benefit.

## 9. Conclusions

As a widely used regional anesthetic, lidocaine not only plays an important role in anesthesia but also exhibits potential applications for the immunoregulation and treatment of inflammatory diseases. Here, we have composed this comprehensive review while highlighting the impacts of lidocaine on both innate and adaptive immune cells, including macrophages, NK cells, monocytes, neutrophils, DCs, eosinophils, mast cells, and T cells. The efficacy of lidocaine has been investigated in different diseases, such as cancer, sepsis, acute lung injury, asthma, organ transplantation, and ischemia–reperfusion injury and diabetes. It can modulate the mRNA expression and protein production of pro-inflammatory or anti-inflammatory cytokines in immune cells via altering molecular signal pathways, including NF-kB, TLR4/p38 MAPK, VSSCs, HIF-1α, TGF-β/Smad3, AMPK-SOCS3, TBK1-IRF7, and G protein-coupled receptors. Therefore, lidocaine may be a prime example of drug repurposing, transforming from a simple local anesthetic to a multifaceted immunomodulatory agent.

**Key Takeaways:** (1) Lidocaine is redundant or dualistic: often pro-inflammatory or anti-tumor in cancer and immunosuppressive in inflammatory disorders. (2) Its mechanisms of action involve the modulation of key immune signaling pathways via VSSC blockade and other interactions. (3) Clinical translation requires careful attention to dosing, timing, and disease context.

We advocate for focused clinical research to integrate lidocaine into immunomodulatory treatment regimens, potentially offering a safe, cost-effective, and widely available therapeutic strategy for a range of immune-mediated or inflammatory diseases.

## Figures and Tables

**Table 1:** Effects of lidocaine on immune cells in vitro.

Cell Type	Origin	Effects	Concentration In Vitro	Ref.
Monocyte	U937	↓ Choline uptake and phosphatidylcholine biosynthesis	0.05–3.2 mM	[11]
	U937	↑ Chromatin condensation, DNA fragmentation, apoptosis, necrosis	6–18 mM	[12]
	THP-1	↓ MCP-1, chemotaxis, peak cytosolic-free calcium	0.3–3000 μM	[13]
	THP-1	↑ p-AMPK, SOCS3; ↓ p-ASK1, p-p38, TF and MMP-2/9 activity	0.01–50 μM	[14]
	Human blood	↓ Tissue factor (TF) and TF activity	20–300 μg/mL	[15]
Macrophage	RAW264.7	↓ M2-TAM, Arg-1; ↑ M1 macrophage, iNOS, TGF-β	1 mM	[16]
	RAW 264.7	↓ iNOS, CAT-2; ↑ GTPCH	5–500 μM	[17]
	RAW 264.7	↓ HMGB1; ↓ translocation of HMGB1 and NF-κB	2–200 μg/mL	[18]
	RAW 264.7	↓ Phagocytic activity	100–1000 μM	[19]
	RAW 264.7	↓ Activation of TLR-4, NF-κB and MAPKs	50 μM	[20]
	RAW 264.7, THP-1	↑ mRNA and protein of IFNα4	300 μM	[21]
	RAW 264.7, THP-1	↓ Nitrite, nitrate and iNOS	5–500 mg/mL	[22]
	THP-1	↑ GDF15, FGF7, HGF, COL4A3, COL8A2, LAMB2, LAMC2, PDGFRA, VEGFA; ↓ CPEB4, SOCS1, SOCS2, SOCS3, DUSP1, TNFAIP3, GATA3	0.1–0.5 mM	[23]
	Human TIICs	↑ CD40, IFN-γ, IL-12, M1 macrophage; ↓ IL-10, TGF-β, IL-35	0.25–1.5 mM	[24]
	Human blood	↓ IL-12; ↑ IL-10, TGF-β, IL-35	0.25–1.5 mM	[24]
	Human blood	↓ IL-1β, TNF-α, IL-6, and IL-8	0.625 mg/mL	[25]
	Human blood	↓ TNF-α	0.5 mM, 1 mM	[26]
	J774A.1	↓ IL-1β, TNF-α, P2X7R; ↓ NLRP3 activation, Lactic dehydrogenase activity; ↓ Na+ inward flow, extracellular K+ level	1–10 μM	[27]
	Mouse PF	↓ Beta-glucuronidase release rate	12 mM	[28]
	Mouse PF	↓ TNF-α, IL-6, GLUT1, HK2, HIF1α; ↓ glycolysis	1–10 μM	[29]
	Mouse PF	↑ Antibody-dependent cytotoxicity and phagocytosis	10 mM	[30]
	Human PF	↓ MCP-1, IL-6 and IL-8	0.1–1.0 mg/mL	[31]
	Guinea pig PF	↓ MIF, cell motility	1–100 mg/mL	[32]
	Guinea pig PF	↓ Immune complexes digestion, immunoglobulin aggregates degradation	5 mM	[33]
	Rat PF	↓ Phagocytosis	0.05–50 μM	[34]
	Rabbit bronchoalveolar lavage fluid	↓ Na^+^/H^+^ exchange, intracellular pH, superoxide production	2.5 mM	[35]
	Rat lung alveolar lavage fluid	↑ Macrophage generation, ↓ cytoplasmic vacuolation	12 mM	[36]
Neutrophil	Human blood	↑ The ability to kill tumor cells	0.1–100 μg	[37]
	Human blood	↓ CD18	0.005–0.5 mg/mL	[38]
	Human blood	↓ Chemotaxis, phagocytosis, O_2_^−^, H_2_O_2_, OH^−^, intracellular calcium ion	20–200 μM	[39]
	Human blood	↑ Early apoptosis	2–4000 μM	[40]
	Human blood	↓ Oxygen radical	1–8 mg/mL	[41]
	Human blood	↓ Oxidative burst, phagocytosis activity, ATP concentration, mitochondrial transmembrane potential; ↑ mitochondrial structural changes and apoptosis	4–400 μM	[42]
	Human blood	↓ O_2_^−^, granule enzymes lysozyme, MPO, bactericidal ability	1.3–2 mg/mL	[43]
	Human blood	↓ CD11b, CD18 and ICAM-1	5–500 μg/mL	[38]
	Pig blood	↓ O_2_^−^, CD11 b/c	1–100 μM	[44]
	Equine blood	↑ Migration and adhesion	0.1–1000 μg/mL	[45]
Eosinophil	Human UCB	↓ Eosinophil count, neurotoxin and peroxidase	0.01–1 mM	[46]
	Human blood	↓ Cellular survival	0.25 mg/mL	[47]
	Human blood	↓ Eosinophil survival, superoxide	0.01–1 mM	[48]
Basophil	Human UCB	↓ Histamine, intracellular Ca^2+^	0.1–10 mM	[49]
NK cell	Human blood	↑ cytotoxicity	0.01–50 μM	[50]
	Human blood	↑ NKG2D and cytolysis	0.01–50 μM	[51]
	Human blood	↓ NK activity	0.02–1 g%	[52]
Mast cell	Mouse bone marrow, Mouse PF	↓ Histamine secretion, intracellular calcium concentration	0.1–10 mM	[49]
	Rat PF	↓ Histamine	6–30 mM	[53]
	Rat PF	↓ Ca++ flux and histamine release	1–100 nM	[54]
	Rat PF	↑ Ca++ flux	10 mM	[54]
	Rat PF	↓ Histamine, Ca influx	0.6–30 mM	[55]
	Rat PF	↑ Histamine, cell lysis	60–100 mM	[53]
DC	Mouse bone marrow	↓ IL-6, TNFα and IL-12	0.2–0.4 mg/mL	[56]
T cell	Mouse Peyer’s patch	↓ IL-2, IL-4, IL-5, IFN-γ; ↓ cell proliferation, activation of p38 and ERK1/2	1–100 μM	[57]
	TIICs	↑ IFN-γ in CD8+ T cells; ↓ IL-10, TGF-β, and IL-35 in CD4+CD25+ T cells; ↑ GPCR signaling, NF-κB activation	0.25–1.5 mM	[24]
	Human blood	↓ IFN-γ in CD8+ T cells, ↑ IL-10, TGF-β, IL-35 in CD4+CD25+ T cells	0.25–1.5 mM	[24]
	Mouse lymph nodes	↓ GATA-3 and cellular proliferation, ↑ cell apoptosis	100–600 μM	[58]
	Jurkat, human blood	↓ IL-2, TNF-α, INF-γ; ↓ nuclear NF-kB, cellular proliferation	0.25–1.5 mM	[59]
	Mouse spleen	↑ FoxP3 expression, Treg differentiation, p-Smad3	0.05–0.8 mM	[60]
	Jurkat	↑ Apoptosis	6 mg/mL	[61]

Upward arrows denote stimulating effects, while downward arrows indicate suppressive effects (TIICs, tumor-infiltrating immune cells; PF, peritoneal fluid; UCB, umbilical cord blood). Common cell lines referenced: RAW 264.7: murine leukemic monocyte–macrophage cell line; THP-1: human monocytic leukemia cell line (often differentiated into macrophage-like cells); Jurkat: human T lymphocyte leukemia cell line; U937: human histiocytic lymphoma cell line (monocytic).

**Table 2:** Effects of lidocaine on immune cells in vivo (animal studies).

Cell Type	Origin	Effects	Concentration In Vivo	Diseases	Ref.
Macrophage	Mouse liver	↑ Phagocytic functions; ↓ ICAM-1, NO, TNF-α, IFN-γ	1 mg/kg, i.v., once every 5 min 4 times	Diabetes	[62]
Neutrophil	Mouse blood	↓ Neutrophil count, edema, pulmonary thrombosis	2, 4, and 8 mg/kg, i.v.	Acute lung injury	[14]
	Canine blood, BALF	↓ Neutrophil count in BALF, ↓ CD11b in blood neutrophils	4 mg/kg, i.v., during surgery and 6 h after surgery	Lung allo-transplantation	[63]
	Equine jejunum	↓ Neutrophil infiltration	1.3 mg/kg, i.v., 15 min after anesthetic induction, 0.05 mg/kg/min during surgery	Intestinal mild ischemia	[64]
	Mouse BALF, lung tissue	↓ Neutrophil cell count	0.25, 0.5, and 1% solution, inh., 30 min after provocation	Asthma	[58]
Eosinophil	Mouse BALF, lung tissue	↓ Eosinophils cell count	0.25, 0.5, and 1% solution, inh.	Asthma	[58]
NK cells	Rat lung lavage fluid	↓ NK activity	1% solution, i.t., 0.75 mL or 3 mL, before lung lavage		[65]
Mast cell	Rat colon mucosa	↓ Mast cell hyperplasia	5 and 10 mg/kg, i.r., once daily for 7 days	Colitis	[66]
	Rat skin	↓ Mast cell count	2% solution, s.c., 2 mL		[67]
T cell	Mouse blood, skin	↑ Foxp3+ cell frequency in skin; ↓ IL17E and IL-4 mRNA, ↑ IFN-γ mRNA in PBMCs	1.5, 3.0 mg/kg/day, i.v., 7 days		[60]

Upward arrows demonstrate stimulating effects, while downward arrows indicate suppressive effects. (i.r., intrarectally; inh., inhalation; top., topically use; s.c., subcutaneous injection; BALF, bronchoalveolar lavage fluid; H3Cit, citrullinated histone H3; MPO, myeloperoxidase).

**Table 3:** Effects of lidocaine on immune cells in vivo (clinical studies).

Cell Type	Origin	Effects	Concentration In Vivo	Diseases	Ref.
Neutrophil	Human blood	↓ Neutrophil arrest, transmigration and p-PKC-θ	1.5 mg/kg, i.v., 100 mg/h for patients > 70 kg, 70 mg/h for patients < 70 kg, 48 h	Septicemia	[68]
	Human blood	↓ H3Cit, MPO	1% solution, i.v., 1.5 mg/kg, at anesthetic induction; 2 mg/kg/h during surgery; 1 mg/kg/h for 24 h after surgery		[69]
	Human blood	↓ H3Cit, MPO	8 mg/kg/h for 15 min before anesthetic induction; 2 mg/kg/h during surgery; 1 mg/kg/h for 24 h after surgery	Non-small cell lung cancer	[70]
	Human blood	↓ MPO, ↑ elastase	1% solution, i.v., 1.5 mg/kg, 10 min after anesthetic induction; 2 mg/kg/h during surgery; 1 mg/kg/h for 24 h after surgery	Prostate cancer	[71]
	Human blood	↓ Circulating NETs	1.5 mg/kg, i.v. during anesthetic induction; 2 mg kg/h during surgery	Pancreatic cancer	[72]
Eosinophil	Human blood	↓ Eosinophil cell count	4% solution, inh., 100 mg/time, 4 times daily for 8 weeks	Asthma	[73]
NK cells	Human blood	↑ NK cells	2% solution, i.v., 1.5 mg/kg, 10 min before anesthetic induction; 2.0 mg/kg/h during surgery		[74]
T cell	Human blood	↑ CD3+ and CD4+ T cells	2% solution, i.v., 1.5 mg/kg, 10 min before anesthetic induction; 2.0 mg/kg/h during surgery	Breast tumor	[74]
	Human blood, skin	↑ Foxp3+ cell frequencies in skin ↓ IL17E mRNA, ↑ IFN-γ, IL-4 and IL-17A mRNA in PBMCs	3 mg/kg/day, i.v., 14 days		[60]

Upward arrows demonstrate stimulating effects, while downward arrows indicate suppressive effects.

## Data Availability

No new data were created or analyzed in this study.

## Abbreviations

ALI, acute lung injury; AMPK, AMP-activated protein kinase; Arg-1, Arginase-1; BALF, bronchoalveolar lavage fluid; CGRP, calcitonin gene-related peptide; CTMC, connective tissue mast cell; DC, dendritic cell; EMT, epithelial–mesenchymal transition; GPCR, G protein-coupled receptor; HIF-1α, hypoxia-inducible factor 1-alpha; HMGB1, high-mobility group box 1; ICAM-1, intercellular adhesion molecule 1; IFN, interferon; IKK, IκB kinase; IL, interleukin; iNOS, inducible nitric oxide synthase; IRF7, interferon regulatory factor 7; IV, intravenous; LPS, lipopolysaccharide; MAPK, mitogen-activated protein kinase; MCP-1, monocyte chemoattractant protein-1; MMC, mucosal mast cell; NET, neutrophil extracellular trap; NF-κB, nuclear factor kappa-light-chain-enhancer of activated B cells; NK cell, natural killer cell; PAD4, peptidylarginine deiminase 4; PBMC, peripheral blood mononuclear cell; PKC-θ, protein kinase C-theta; RCT, randomized controlled trial; ROS, reactive oxygen species; Smad3, SMAD family member 3; SOCS3, suppressor of cytokine signaling 3; TAM, tumor-associated macrophage; TBK1, TANK-binding kinase 1; TCR, T cell receptor; TF, tissue factor; TGF-β, transforming growth factor-beta; Th cell, T helper cell; TIIC, tumor-infiltrating immune cell; TLR4, toll-like receptor 4; Treg, regulatory T cell; VSSC, voltage-sensitive sodium channel.
